# Gd^3+^-Doping Effect on Upconversion Emission of NaYF_4_: Yb^3+^, Er^3+^/Tm^3+^ Microparticles

**DOI:** 10.3390/ma13153397

**Published:** 2020-07-31

**Authors:** Aleksandra A. Vidyakina, Ilya E. Kolesnikov, Nikita A. Bogachev, Mikhail Y. Skripkin, Ilya I. Tumkin, Erkki Lähderanta, Andrey S. Mereshchenko

**Affiliations:** 1 Saint-Petersburg State University, 7/9 Universitetskaya Emb., St. 199034 Petersburg, Russia; vidyakina.aleksandra@mail.ru (A.A.V.); ilya.kolesnikov@spbu.ru (I.E.K.); allanfrack@yandex.ru (N.A.B.); skripkin1965@yandex.ru (M.Y.S.); i.i.tumkin@spbu.ru (I.I.T.); 2 Sirius University of Science and Technology, 1 Olympic Ave, 354340 Sochi, Russia; 3 LUT University, Skinnarilankatu 34, 53850 Lappeenranta, Finland; erkki.lahderanta@lut.fi

**Keywords:** upconversion, luminescence, microcrystals, hydrothermal synthesis, rare earth

## Abstract

*β*-NaYF_4_ microcrystals co-doped with Yb^3+^, Er^3+^/Tm^3+^, and Gd**^3+^** ions were synthesized via a hydrothermal method using rare-earth chlorides as the precursors. The SEM and XRD data show that the doped *β*-NaYF_4_ form uniform hexagonal prisms with an approximate size of 600–800 nm. The partial substitution of Y by Gd results in size reduction of microcrystals. Upconversion luminescence spectra of microcrystals upon 980 nm excitation contain characteristic intra-configurational ff bands of Er^3+^/Tm^3+^ ions. An addition of Gd^3+^ ions leads to a significant enhancement of upconversion luminescence intensity with maxima at 5 mol % of dopant.

## 1. Introduction

Rare earth-based materials are known to demonstrate efficient upconversion properties and are able to transform near-infrared (NIR) light to visible or even UV light via multiphoton processes [1,2,3]. NaYF_4_ doped by rare earth ions is one of the most efficient upconversion phosphors among numerous luminescent materials due to the low phonon energy of host lattice, which reduces the amount of nonradiative transitions [4,5]. Lanthanide elements have attracted intense attention in recent years in numerous fields, such as photodynamic therapy [6,7], flat-panel displays [8], solid-state lasers [9,10,11], bio-imaging [4,12,13], and biosensing [14].

NaYF_4_: Yb^3+^, Tm^3+^/Er^3+^ upconversion microcrystals are known to have the best luminescence property of all fluorescent materials [15]. Different methods for the synthesis of NaYF_4_: Yb^3+^, Tm^3+^/Er^3+^ have been recently reported, including hydrothermal and solvothermal methods [16,17,18,19]. Using various synthetic approaches, particles of different sizes can be obtained. In solvothermal synthesis with oleic acid/octadecene solvent, hexagonal nanoparticles of a small size (<100 nm) are obtained. Microcrystals of a larger size (>500 nm), which can be fabricated by hydrothermal synthesis, usually have higher luminescence intensity. The Tm^3+^ and Er^3+^ ions act as optical active centers; the Yb^3+^ ion is a sensitizer that absorbs NIR light and then transfers energy to Tm^3+^ or Er^3+^.

In our work, we partially substituted Yb^3+^ by Gd^3+^ ions in NaYF_4_: Yb^3+^, Tm^3+^/Er^3+^ materials to improve upconverting properties. It was previously demonstrated that Gd^3+^ co-doping improves the luminescent properties of rare earth-based materials [17,20]. By introducing Gd^3+^ ions into the NaYF_4_ crystal lattice, it is possible to change local symmetry, thus increasing the probability of energy transfer processes, which could increase luminescence intensity. We studied the structure and upconverting luminescent properties of NaYF_4_: Gd^3+^/Yb^3+^/Tm^3+^ and NaYF_4_: Gd^3+^/Yb^3+^/Er^3+^ microparticles synthesized via a hydrothermal method. We found that co-doping of 5% Gd^3+^ ions in NaYF_4_: Yb^3+^, Tm^3+^/Er^3+^ increases the upconversion luminescence intensity in the visible range by 2–5 times upon 980 nm excitation.

## 2. Materials and Methods

Anhydrous chlorides of the rare earth elements (YCl_3_, ErCl_3_, GdCl_3_, YbCl_3_, and TmCl_3_, 99.999%) were purchased from Chemcraft (Russia), NaOH, NH_4_F, sodium citrate, and ethanol were purchased from Sigma-Aldrich Pty Ltd. (Germany), and used without additional purification.

Microcrystalline *β*-NaYF_4_ samples co-doped with Yb^3+^, Er^3+^, Tm^3+^, and Gd**^3+^** were synthesized by the hydrothermal method using citric acid as a stabilizing agent. We redesigned the previously reported hydrothermal method of synthesis [16,17,18,19]. In the typical synthesis, yttrium, ytterbium(III), gadolinium(III), and thulium(III)/erbium(III) chlorides (total amount of rare earth chlorides was 0.75 mmol) with 3 mmol of citric acid were dissolved in distilled water to obtain 5 mL solution in total. Chlorides of rare earth elements were taken in stoichiometric amounts. Then, 2.5 mL of aqueous solution containing 9 mmol of NaOH was added to the flask of the previous solution. After vigorous stirring for 30 min, 8 mL of aqueous solution containing 11 mmol of NaOH and 11 mmol of NH_4_F was introduced into the above solution. The solution was maintained after vigorous stirring for 30 min at room temperature before being transferred to a Teflon-lined autoclave with an internal volume of 20 mL. The hydrothermal syntheses were conducted in an electric oven at 180 °C for 24 h. After that, the precipitate was separated from the reaction mixture by centrifugation, washed with ethanol and deionized water, and dried at 60 °C for 24 h. The desired microstructure materials were obtained in a form of white powders.

Dopant concentration, particle size, and crystallite phase are known to significantly influence the efficiency of upconversion luminescence [21,22]. Earlier studies demonstrated that the Yb^3+^ optimal concentration is about 20 at % [23,24,25]. Our preliminary experiments demonstrated that in NaY_0.8-y_Yb_0.20_Tm_y_F_4_ and NaY_0.8-z_Yb_0.20_Er_z_F_4_, the optimal concentration of Tm^3+^ and Er^3+^ is in the range of 0.5–1 at %, which agrees with earlier studies where the optimal dopant concentration for Tm^3+^ and Er^3+^ ions varied from 1% to 2% [18,20,22,23]. Therefore, in this work, we synthesized and studied the two Gd^3+^ co-doping series of upconverting microcrystals with 1% Tm^3+^/Er^3+^ concentration: NaY_0.79−x_Yb_0,20_Tm_0.01_Gd_x_F_4_ and NaY_0.79−x_Yb_0,20_Er_0.01_Gd_x_F_4_ (x = 0–0.2).

The morphologies of microstructures of the synthesized samples were characterized using scanning electron microscopy (SEM) with a Zeiss Merlin electron microscope (Zeiss, Germany) with energy-dispersive X-ray spectroscopy (EDX) module (Oxford Instruments INCAx-act, UK) and confirmed by atomic force microscopy (AFM) using a Nanoeductor II microscope (NT-MDT Spectrum Instruments, Moscow, Russia); the AFM measurements were performed in a semi-contact regime. X-ray powder diffraction (XRD) measurements were performed on a D2 Phaser (Bruker, Billerica, MA, USA) X-ray diffractometer using (Cu Kα radiation, λ = 1.54056 Å) radiation. The upconversion luminescence emission spectra were recorded with an Fluorolog-3 fluorescence spectrometer (Horiba Jobin Yvon, Japan) with diode laser (wavelength 980 nm, power 320 mW, and beam diameter 2 mm) as an excitation source for upconversion luminescence. Lifetime measurements were performed with the same spectrometer using pulsed Xe lamp (pulse duration 3 µs).

## 3. Results and Discussion

### 3.1. Crystal Structure and Morfology

**Figure 1:**
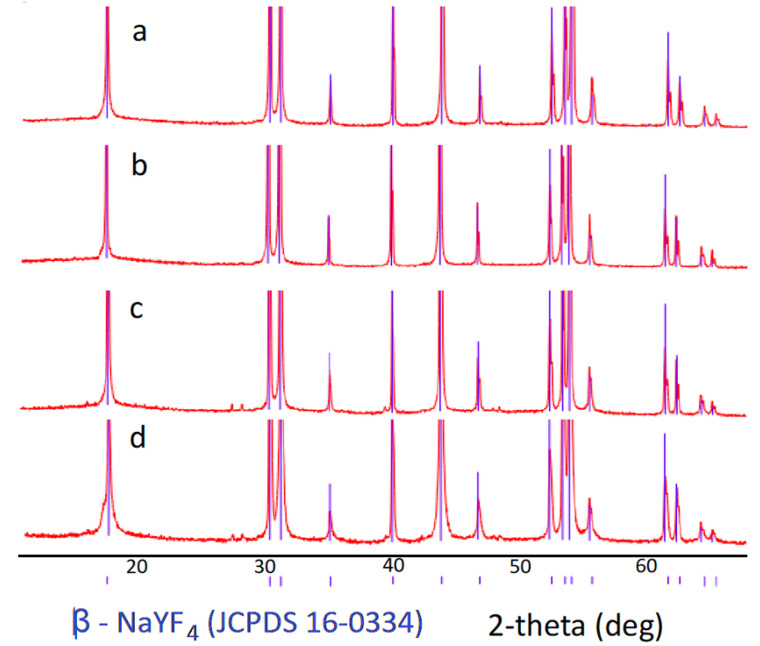
XRD patterns of the samples: (**a**) β-NaYF_4_: 20% Yb, 1% Tm, (**b**) β-NaYF_4_: 20%Yb, 1% Er, (**c**) β-NaYF_4_: 20% Yb, 1% Tm, 5% Gd, and (**d**) β-NaYF_4_: 20% Yb, 1% Er, 5% Gd. Blue lines show standard values for pure hexagonal β-NaYF_4_.

NaYF_4_ exists in two phases [5,26,27,28]: cubic α-NaYF_4_ phase and hexagonal β-NaYF_4_ phase. The upconverting efficiency of the hexagonal phase NaYF_4_: Yb,Tm/Er materials are significantly higher [19,29,30]. X-ray powder diffraction (XRD) patterns of the synthesized samples are given in Figure 1.

The diffraction maxima positions of all our samples matched the standard values for pure hexagonal β-NaYF_4_ (JCPDS No. 16-0334). No diffraction peaks attributed to impurities were observed. We found that the addition of Gd did not lead to a phase transformation. The XRD data of all the samples were the same; therefore, only several XRD patterns are given here for simplicity.

**Figure 2:**
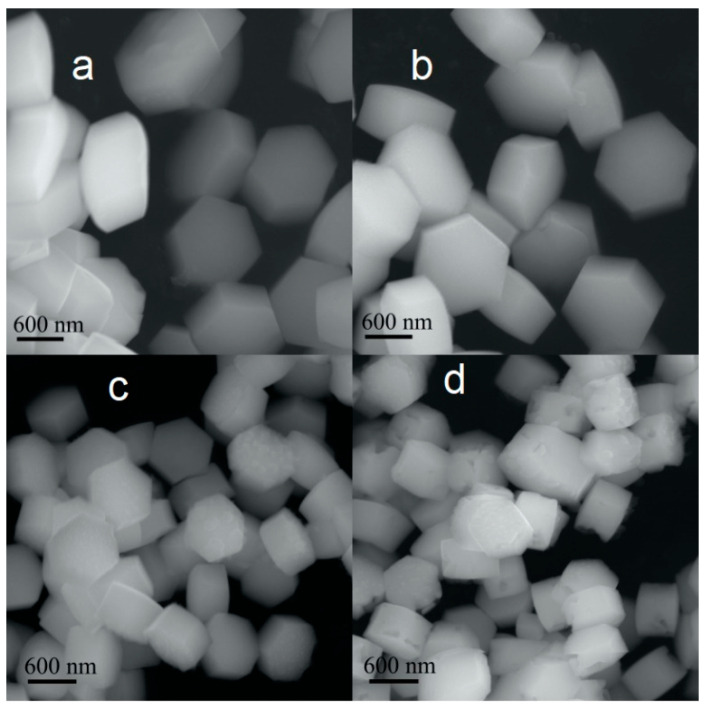
SEM images of the samples: (**a**) β-NaYF_4_: 20% Yb, 1% Tm, (**b**) β-NaYF_4_: 20% Yb, 1% Er, (**c**) β-NaYF_4_: 20% Yb, 1% Tm, 5% Gd, and (**d**) β-NaYF_4_: 20% Yb, 1% Er, 5% Gd. All the samples were synthesized with the same reaction time (24 h).

Scanning electron microscope (SEM) was used to analyze the shape and size of the microcrystals. SEM images of various composition microcrystals are shown in Figure 2.

**Figure 3:**
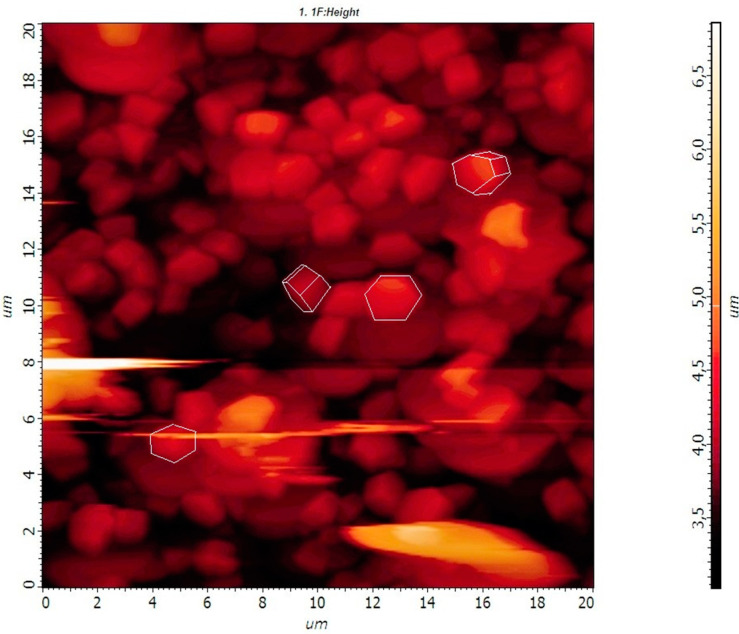
AFM image of the sample β-NaYF_4_: 20%Yb, 1% Tm, 5% Gd.

All the samples consisted of sub-micron-sized uniform hexagonal prism-shaped particles (Figure 2a–d). The morphology of the microcrystals obtained by SEM agreed with that obtained by AFM (Figure 3).

**Figure 4:**
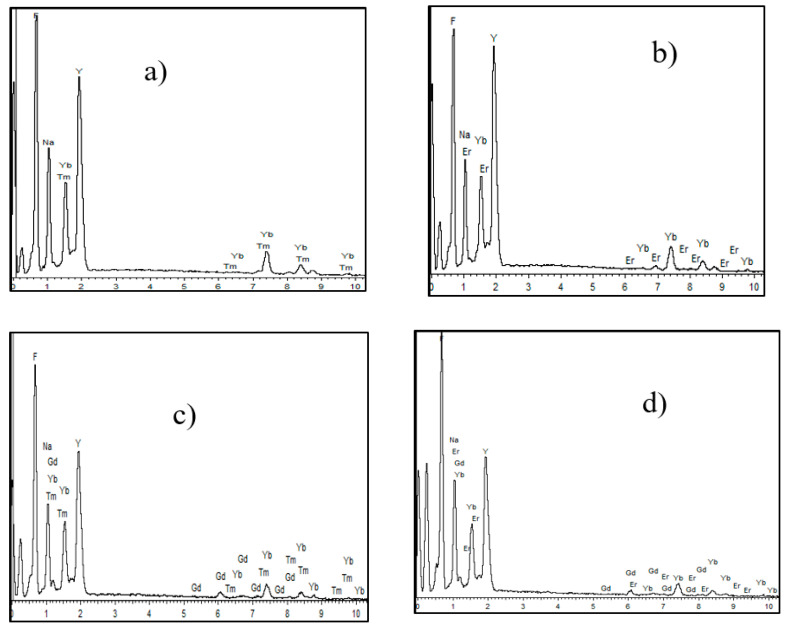
EDX spectra of the samples: (**a**) β-NaYF_4_: 20%Yb, 1% Tm, (**b**) β-NaYF_4_: 20%Yb, 1% Er, (**c**) β-NaYF_4_: 20%Yb, 1% Tm, 5% Gd, and (**d**) β-NaYF_4_: 20%Yb, 1% Er, 5% Gd.

The microcrystals without Gd^3+^ dopant (Figure 2a,b) had a uniform morphology and an average length along the diagonal direction of about 800 nm. Notably, the addition of the Gd^3+^ dopant (Figure 2c,d) led to a decrease in the size of the microcrystals that is clearly seen from the SEM images. Addition of Gd^3+^ ions in NaYF_4_: Yb, Tm/Er also resulted in the formation of surface defects (Figure 2c,d), such as cracks and chips. The size of the crystals is probably guided by crystal growth rates. Earlier studies demonstrated that substitution of yttrium ion by larger gadolinium(III) ion (ionic radii of Y^3+^ and Gd^3+^ are 1.159 and 1.193 Å, respectively) results in an increase in the electron charge density of the crystal surface [31,32]. Therefore, the larger electron charge density in the Gd^3+^-containing crystal nucleus slows the diffusion of negatively charged fluoride ions, which leads to a reduction in the crystal growth rate and a smaller final size of Gd^3+^ co-doped microcrystals. Furthermore, the difference in charge density inside the crystal can result in a minor change of local symmetry of rare earth ions and surface structural defects. The composition of microcrystals was roughly estimated by energy dispersive X-ray analysis (EDX). The EDX spectra (Figure 4) indicated the presence of all elements (Y, Yb, F, Na, Gd, and Er/Tm) in the synthesized materials.

### 3.2. Luminescence Properties

**Figure 5:**
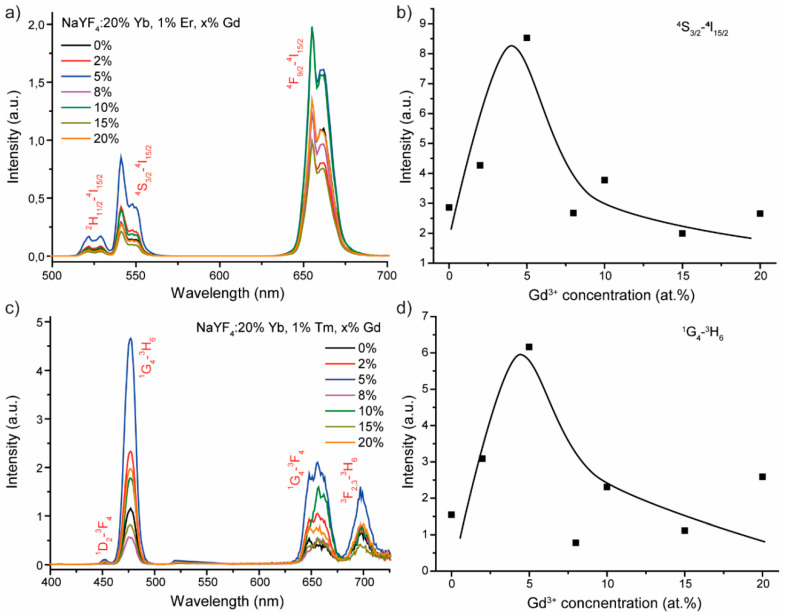
(**a**) Upconversion luminescence of NaYF_4_: 20% Yb, 1% Er microparticles with different Gd^3+^ concentrations, (**b**) the dependence of green emission (541 nm) intensity on the Gd^3+^ amount, (**c**) upconversion luminescence of NaYF_4_: 20% Yb, 1% Tm microparticles with different Gd^3+^ concentration, and (**d**) the dependence of blue emission (477 nm) intensity on the Gd^3+^ amount.

Upconversion spectra of NaYF_4_: 20% Yb, 1% Er microcrystals with different Gd^3+^ concentration upon 980 nm excitation are shown in Figure 5a.

Emission spectra measured in the spectral range 500–700 nm consisted of characteristic sharp lines corresponding to the intra-configurational 4f transitions of erbium ions. The observed emission peaks are assigned to ^2^H_11/2_–^4^I_15/2_ (522 and 529 nm), ^4^S_3/2_–^4^I_15/2_ (541 and 548 nm), and the most prominent ^4^F_9/2_–^4^I_15/2_ (655 and 661 nm) transitions [17,33]. Note, concentration of Gd^3+^ ions non-monotonically affected emission intensity. Optimal Gd^3+^ co-doping concentration was 5% for green emission, whereas red emission showed equal intensities for 5% and 10% Gd^3+^ co-doped samples. An example of Gd^3+^ concentration dependence of emission intensity (^4^S_3/2_–^4^I_15/2_ integral intensity) of NaYF_4_: 20% Yb, 1% Er, Gd phosphors is shown in Figure 5b. At first, luminescence intensity increased along with Gd^3+^ ions growth, reaching a maximum at 5%. Further increase in Gd^3+^ ions resulted in concentration quenching.

Figure 5c presents upconversion spectra of NaYF_4_:20% Yb, 1% Tm powders with different Gd concentration upon 980 nm excitation. The obtained emission spectra include the following transitions: ^1^D_2_–^3^F_4_ (452 nm), ^1^G_4_–^3^H_6_ (477 nm), ^1^G_4_–^3^F_4_ (648 nm and 656 nm), and ^3^F_2,3_–^3^H_6_ (697 nm) [32,34]. Similar to NaYF_4_: Yb, Er, Gd samples, the addition of gadolinium ions in NaYF_4_: Yb, Tm phosphors significantly affected emission intensity. Evolution of ^1^G_4_–^3^H_6_ integral intensity as a function of Gd^3+^ concentration is presented in Figure 5d. Analyzing the obtained experimental data, we concluded that the best luminescence intensity enhancement was achieved for 5% Gd^3+^-co-doped powder.

Upconversion intensity enhancement by Gd^3+^ co-doping of NaYF_4_: Yb, Er or NaYF_4_: Yb, Tm is usually explained by host phase transition from cubic to hexagonal, which would significantly improve luminescence intensity [17,35]. However, in our case, hexagonal phase formed even in the case of Gd^3+^-free powders. Introduction of Gd^3+^ ions in the NaYF_4_ host leads to the formation of crystal lattice defects, as shown in Figure 2c,d, which change the symmetry of the surroundings of ytterbium, thulium, and erbium ions. Thereby, energy transfer processes and/or radiative transitions become more possible from the symmetry point of view, which leads to an increase in luminescence intensity [36]. This suggestion is confirmed by comparison of Gd^3+^ (r = 1.193 Å) and Y^3+^ (r = 1.159 Å) ionic radii [31] displaying possible appearance of crystal lattice defects as a result of gadolinium co-doping. The addition of a large amount of Gd^3+^ ions reduced Er^3+^ and Tm^3+^ luminescence due to two co-directional processes. Firstly, large numbers of crystal lattice defects enhance nonradiative decay rate, which decreases luminescence intensity. Secondly, high Gd^3+^ co-doping concentration promotes energy transfer from high excited states of thulium and erbium to gadolinium ions [36].

**Figure 6:**
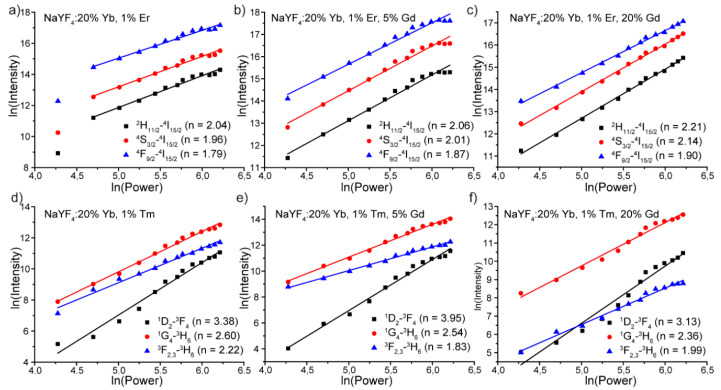
Dependence of integral upconversion luminescence on laser power of (**a**) NaYF_4_: 20% Yb, 1% Er; (**b**) NaYF_4_: 20% Yb, 1% Er, 5% Gd; (**c**) NaYF_4_: 20% Yb, 1% Er, 20% Gd; (**d**) NaYF_4_: 20% Yb, 1% Tm; (**e**) NaYF_4_: 20% Yb, 1% Tm, 5% Gd; and (**f**) NaYF_4_: 20% Yb, 1% Tm, 20% Gd microparticles.

To study the mechanism of upconversion processes in NaYF_4_: Yb, Er, Gd and NaYF_4_: Yb, Tm, Gd phosphors, we measured the emission intensity dependence on pump power. The upconversion emission intensity (I_UC_) increased proportionally to the pumping power (*p*) of the excitation source according to I_UC_–P^n^, where n is the number of photons that pump the population in a particular energy level [26,36]. Therefore, n, the number of photons involved in the upconversion emission, can be obtained from the logarithmic plot of the integral emission intensity vs. the incident laser power. Figure 6a–c show the plot of the integral emission intensity of the green and red emission lines as a function of the pump laser power for NaYF_4_: Yb, Er, Gd powders.

All experimental data can be perfectly fitted using linear function with the slopes of 1.79–2.22 on a log-log plot giving n ≈ 2. We concluded that the observed ^2^H_11/2_–^4^I_15/2_, ^4^S_3/2_–^4^I_15/2_ and ^4^S_3/2_–^4^I_15/2_ transitions in NaYF_4_: Yb, Er, and Gd samples originated from two-photon process [37] irrespective of Gd^3+^ co-doping concentration. Figure 4d–f present integral emission intensity of the blue and red emission lines as a function of the pump laser power for NaYF_4_: Yb, Tm, and Gd powders. Similar to NaYF_4_: Yb, Er, and Gd samples, the amount of Gd^3+^ ions did not affect the number of photons needed to excite certain transition. ^1^D_2_–^3^F_4_, ^1^G_4_–^3^H_6_, and ^3^F_2,3_–^3^H_6_ transitions require absorption of 4, 3, and 2 photons, respectively.

**Figure 7:**
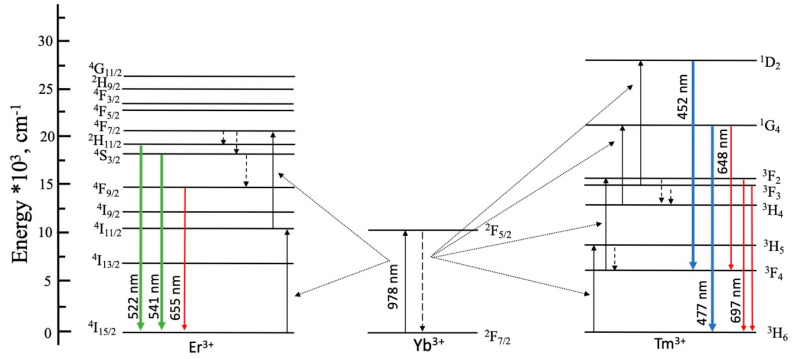
Schematic energy level diagrams of Yb^3+^, Tm^3+^, Er^3+^, and possible upconversion emission processes upon 980 nm excitation.

Based on the obtained experimental data, the energy level diagrams of Yb^3+^, Er^3+^, and Tm^3+^ ions, as well as the possible energy transfer mechanisms for upconversion emissions in NaYF_4_ host upon 980 nm excitation, are shown in Figure 7.

Gd^3+^ ions have a very large energy gap between ground ^8^S_7/2_ and first-excited ^6^P_7/2_ states (>30,000 cm^−1^). Therefore, gadolinium ions could participate in energy transfer processes in highly-doped NaYF_4_: Yb, Er, Gd and NaYF_4_: Yb, Tm, Gd samples. Large numbers of Gd^3+^ ions promote quenching of Er^3+^ and Tm^3+^ emission through depopulation of their upper excited levels by following energy transfers: ^4^G_9/2_–^4^I_15/2_ (Er^3+^):^8^S_7/2_–^6^I_J_ (Gd^3+^), ^4^G_7/2_ and ^2^K_13/2_–^4^I_15/2_ (Er^3+^):^8^S_7/2_–^6^P_J_ (Gd^3+^); ^3^P_0,1,2_–^3^H_6_ (Tm^3+^):^8^S_7/2_–^6^I_J_ (Gd^3+^); and ^1^I_6_–^3^H_6_ (Tm^3+^):^8^S_7/2_–^6^P_7/2,5/2_ (Gd^3+^) [36,37,38]. When upper energy levels of Er^3+^ and Tm^3+^ are populated (even minor amounts), there are two possibilities of energy dissipation: (1) internal conversion to lower levels of Er^3+^ and Tm^3+^ followed by luminescence and (2) the energy transfer to Gd^3+^. Therefore, a large concentration of Gd^3+^ ions significantly decreases the population of upper energy levels of Er^3+^ and Tm^3+^, leading to some decrease in the population of the states from which luminescence occurs.

**Figure 8:**
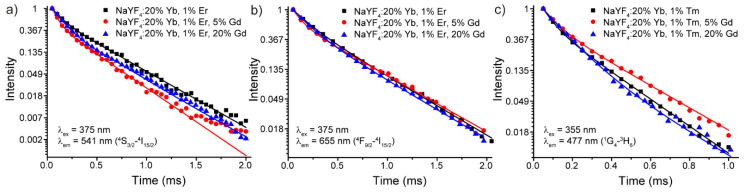
Decay curves of NaYF_4_: 20% Yb, 1% Er/1% Tm, Gd microparticles monitored for (**a**) ^4^S_3/2_–^4^I_15/2_ (541 nm), (**b**) ^4^F_9/2_–^4^I_15/2_ (655 nm), and (**c**) ^1^G_4_–^3^H_6_ (477 nm) transitions.

To provide a more detailed study of the Gd^3+^ co-doping effect on luminescence properties of NaYF_4_: Yb, Er, Gd and NaYF_4_: Yb, Tm, and Gd powders, we carried out kinetics measurements. Decay curves of NaYF_4_: 20% Yb, 1% Er/1% Tm (without Gd^3+^ co-doping); NaYF_4_: 20% Yb, 1% Er/1% Tm, 5% Gd (the most prominent sample); and NaYF_4_: 20% Yb, 1% Er/1% Tm, 20% Gd (highly Gd^3+^ co-doped sample) were recorded (Figure 8).

Notably, the kinetics studies were performed upon Stokes excitation (*λ_ex_* = 375 and 355 nm for Er^3+^ and Tm^3+^-doped phosphors, respectively). ^4^S_3/2_–^4^I_15/2_ (541 nm) and ^4^F_9/2_–^4^I_15/2_ (655 nm) transitions were monitored in Er^3+^-doped samples, and ^1^G_4_–^3^H_6_ (477 nm) transition was measured in Tm^3+^-doped powders. All experimental decay curves displayed non-single exponential behavior and two exponential models were applied for fitting. Average luminescence lifetime (*τ_av_*) was calculated according to the following equation to simplify comparison [35,36]:

(1) $$\tau_{\mathrm{av}}=\frac{A_{1}\tau_{1}^{2}+A_{2}\tau_{2}^{2}}{A_{1}\tau_{1}+A_{2}\tau_{2}}$$

where A_1_ and A_2_ are pre-exponential constants, and τ_1_ and τ_2_ are fitting lifetimes (Table S1, Supplementary Materials). The calculated lifetimes of NaYF_4_: Yb, Er, Gd and NaYF_4_: Yb, Tm, Gd powders are listed in Table 1. The obtained lifetimes are in agreement with the previous studies, where the typical luminescence lifetimes are in the range of 0.1–0.5 ms depending on the morphology and composition [38,39,40,41].

The introduction of 5% Gd^3+^ ions affected the average lifetime more profoundly compared with 20% Gd^3+^ doped sample, which is consistent with earlier the observed concentration dependence of emission intensity. Nonmonotonic lifetime changes in Er^3+^ and Tm^3+^-doped phosphors may be due to different mechanisms of Gd doping on the monitored emission transitions.

## 4. Conclusions

We synthesized hexagonal NaYF_4_ microcrystals co-doped with different rare earth ions Yb^3+^, Tm^3+^/Er^3+^, and Gd^3+^ via a hydrothermal method: NaY_0.79−х_Yb_0,20_Er_0.01_Gd_x_F_4_ and NaY_0.79−х_Yb_0,20_Tm_0.01_Gd_x_F_4_ (x = 0–0.2). The size of the synthesized particles was determined to be about 800 nm for NaY_079_Yb_0,20_Tm_0.01_F_4_ and NaY_0.79_Yb_0,20_Er_0.01_F_4_, and about 600 nm for NaY_0.79−х_Yb_0,20_Er_0.01_Gd_x_F_4_ and NaY_0.79−х_Yb_0,20_Tm_0.01_Gd_x_F_4._ The decrease in particle size when co-doped with Gd^3+^ ions is explained by the slower crystal growth rates due to an increase in the electron charge density of the crystal surface in Gd^3+^-co-doped microcrystals. XRD showed that all the samples consisted of hexagonal phase and the addition of Gd^3+^ did not lead to phase transformation.

All synthesized materials demonstrated prominent upconversion luminescence upon 980 nm excitation. The addition of gadolinium enhances upconversion luminescence. This is probably associated with the appearance of crystal lattice defects, which change the symmetry of the surroundings of ytterbium, thulium, and erbium ions. Thus, energy transfer processes and/or radiative transitions become enabled from the symmetry point of view, which results in an increase in luminescence intensity. Larger numbers of Gd^3+^ ions promote quenching of Er^3+^ and Tm^3+^ emission through depopulation of their upper excited levels. We found an optimal composition of the particles for the maximum intensity luminescence: NaYF_4_: 20% Yb, 1% Er, 5% Gd and NaYF_4_: 20% Yb, 1% Tm, 5% Gd. Possible energy transfer mechanisms for upconversion emissions in NaYF_4_ host co-doped with different rare earth ions Yb, Tm, Er, and Gd upon 980 nm excitation were proposed.

## Figures and Tables

**Table 1:** Average luminescence lifetimes of Yb, 1% Er/1% Tm, Gd microparticles.

Sample	Transition	τ_av_, ms
NaYF_4_: 20% Yb, 1% Er	^4^S_3/2_–^4^I_15/2_ (541 nm)	0.34
NaYF_4_: 20% Yb, 1% Er, 5% Gd		0.25
NaYF_4_: 20% Yb, 1% Er, 20% Gd		0.33
NaYF_4_: 20% Yb, 1% Er	^4^F_9/2_–^4^I_15/2_ (655 nm)	0.46
NaYF_4_: 20% Yb, 1% Er, 5% Gd		0.48
NaYF_4_: 20% Yb, 1% Er, 20% Gd		0.44
NaYF_4_: 20% Yb, 1% Tm	^1^G_4_–^3^H_6_ (477 nm)	0.19
NaYF_4_: 20% Yb, 1% Tm, 5% Gd		0.23
NaYF_4_: 20% Yb, 1% Tm, 20%Gd		0.18

## Supplementary Materials

The following are available online at https://www.mdpi.com/1996-1944/13/15/3397/s1, Figure S1: (a) The dependence of red emission (655 nm) intensity on the Gd^3+^ amount in NaYF_4_: 20% Yb, 1%, Er, Gd microparticles; (b) the dependence of red emission (648 nm) intensity on the Gd^3+^ amount in of NaYF_4_: 20% Yb, 1%, Tm, Gd microparticles, Table S1: Luminescence lifetimes of Yb, 1% Er/1% Tm, Gd microparticles.
