# Supplementary material for: Gd3+-Doping Effect on Upconversion Emission of NaYF4: Yb3+, Er3+/Tm3+ Microparticles
Source: Materials (Basel). 2020 Jul 31;13(15):3397. doi: 10.3390/ma13153397 (PMC7435447; doi:10.3390/ma13153397)
Supplement: Supplementary file 1 [file materials-13-03397-s001.pdf]

materials

Supplementary materials

Gd^{3+}-Doping Effect on Upconversion Emission of $NaYF_4$: Yb^{3+}, Er^{3+}/Tm^{3+} Microparticles

Table S1. Luminescence lifetimes of Yb, 1% Er/1% Tm, Gd microparticles.

Sample	Transition	A_1	τ_1, ms	A_2	τ_2, ms	τ_{av}, ms
$NaYF_4$:20% Yb, 1% Er	$^4S_{3/2}$–$^4I_{15/2}$ (541 nm)	0.608	0.094	0.732	0.390	0.34
$NaYF_4$:20% Yb, 1% Er, 5%Gd		0.576	0.323	1.115	0.064	0.25
$NaYF_4$:20% Yb, 1% Er, 20%Gd		1.001	0.128	0.363	0.475	0.33
$NaYF_4$:20% Yb, 1% Er	$^4F_{9/2}$–$^4I_{15/2}$ (655 nm)	0.409	0.168	0.767	0.507	0.46
$NaYF_4$:20% Yb, 1% Er, 5%Gd		0.597	0.096	0.709	0.534	0.48
$NaYF_4$:20% Yb, 1% Er, 20%Gd		0.542	0.172	0.653	0.512	0.44
$NaYF_4$:20% Yb, 1% Tm	$^1G_4$–$^3H_6$ (477 nm)	0.949	0.058	0.745	0.228	0.19
$NaYF_4$:20% Yb, 1% Tm, 5%Gd		0.726	0.276	0.777	0.074	0.23
$NaYF_4$:20% Yb, 1% Tm, 20%Gd		0.317	0.276	1.105	0.119	0.18

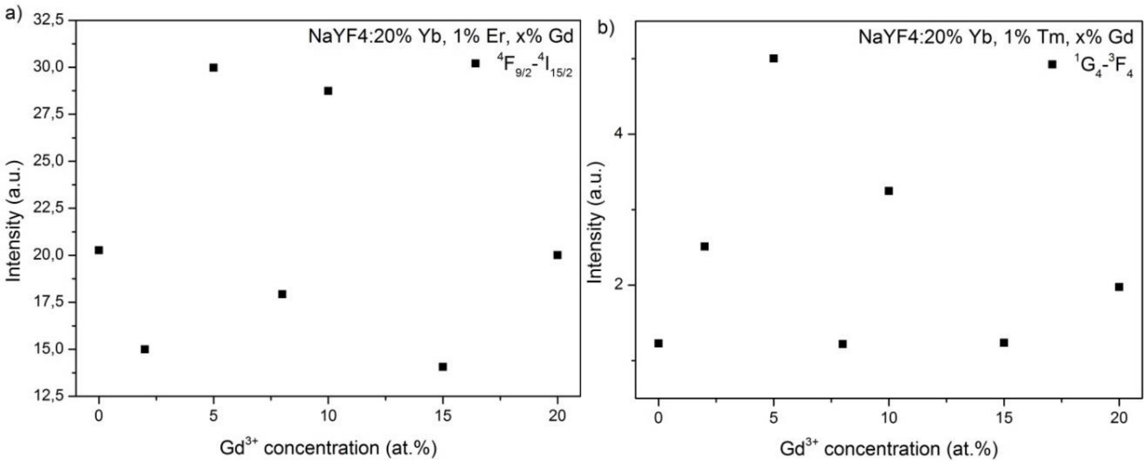

Figure S1. (**a**) The dependence of red emission (655 nm) intensity on the Gd^{3+} amount in $NaYF_4$: 20% Yb, 1%, Er, Gd microparticles; (**b**) the dependence of red emission (648 nm) intensity on the Gd^{3+} amount in of $NaYF_4$: 20% Yb, 1%, Tm, Gd microparticles.
